# Anti-interleukin-23 treatment linked to improved *Clostridioides difficile* infection survival

**DOI:** 10.1080/19490976.2025.2480195

**Published:** 2025-03-21

**Authors:** Gregory R. Madden, Robert Preissner, Saskia Preissner, William A. Petri

**Affiliations:** a Division of Infectious Diseases & International Health, Department of Medicine, University of Virginia School of Medicine, Charlottesville, VA, USA; b Office of Hospital Epidemiology/Infection Prevention & Control, UVA Health, Charlottesville, VA, USA; c Science-IT and Institute of Physiology, Charité—Universitätsmedizin Berlin, Corporate Member of Freie Universität Berlin, Humboldt-Universität zu Berlin, and Berlin Institute of Health, Berlin, Germany

**Keywords:** *Clostridioides difficile*, *C. difficile* infection, monoclonal antibody, interleukin-23, IL-23, Th17 immunity, retrospective study

## Abstract

*Clostridioides difficile* is a leading cause of healthcare-associated infection, and an unacceptably high proportion of patients with *C. difficile* infection die despite conventional antibiotic treatment. Host-directed immunotherapy has been proposed as an ideal treatment modality for *C. difficile* infection to mitigate the underlying toxin-mediated pathogenic immune response while sparing protective gut microbes. Interleukin-23 monoclonal antibody inhibitors are used extensively to control pro-inflammatory Th17 immune pathways in psoriasis and inflammatory bowel disease that are similarly important during *C. difficile* infection. We used a large retrospective electronic health record database to test the hypothesis that hospitalized patients with *C. difficile* infection who are on anti-IL-23 treatment will have improved survival compared to patients without anti-IL-23. A total of 9,301 anti-IL-23 patients had significantly lower probability of all-cause death within 30 d (0.54%) compared with 1:1 propensity-matched control patients (3.1%). IL-23 inhibition is a promising adjunct to *C. difficile* treatment, and further clinical trials repositioning anti-IL-23 monoclonal antibodies from psoriasis and inflammatory bowel disease to *C. difficile* infection are warranted.

## Introduction

*Clostridioides difficile* is one of the leading healthcare-associated pathogens associated with unexpected in-hospital death.^1^ *C. difficile* produces a diarrheal toxin that stimulates a strong pathogenic immune response, resulting in death in 5% or more of cases.^2^ Recent advances in microbiota and anti-toxin therapies given after recovery from *C. difficile* infection are proven to restore a healthy gut microbiome and prevent recurrent infection but do not address early severe outcomes, including death.

Antibiotics in *C. difficile* infection (including those used against *C. difficile*) are a double-edged sword because they disrupt the protective gut microbiota and predispose to further *C. difficile* acquisition, growth, and reinfection. Clinical options for augmenting treatment in severe or refractory *C. difficile* infection remain extremely limited, and each lacks strong evidence to support their efficacy: 1) changing or combining anti-*C. difficile* antibiotics, 2) changing antibiotic administration route (per rectum, via ileostomy), 3) early fecal microbiota transplant (rarely done during acute infection due to limited evidence and logistical barriers), and/or 4) intestinal surgery.^3^

IL-23 signaling induces colitis in mouse models of inflammatory bowel disease,^4^ and, similarly, interleukin-23 (IL-23)-mediated Th17 immunity plays a pivotal role in neutrophil activation and severity of *C. difficile* infection.^5^ IL-23-positive immune cells infiltrate the human colon during *C. difficile* colitis,^6^ and higher IL-23 levels correlate with inflammasome activation^7^ and worsening disease severity.^8^ IL-23 neutralization in mice prevents *C. difficile* infection mortality,^6^ and IL-23 inhibitors have been proposed as a promising candidate for host-directed immunotherapy in *C. difficile* infection.^9^

Beginning with ustekinumab (FDA approved in 2009), monoclonal antibodies against IL-23 (specifically, the pro-inflammatory p19 subunit, IL-23p19) have become a mainstay of treatment for psoriasis and inflammatory bowel disease. Here, we report a retrospective, propensity-matched case––control study of *C. difficile* infection-associated 30-d mortality in patients receiving anti-IL-23 treatment compared to patients without anti-IL-23.

## Method

Data were collected from the COVID-19 Research Network provided by TriNetX, which includes 94 healthcare organizations in 11 countries. The time window used for this analysis was between 20 y and 30 d prior to the query on October 9, 2024. Hospitalized *C. difficile* infection cases were defined as inpatient encounters with a positive *C. difficile* test (polymerase chain reaction or enzyme immunoassay) and/or International Classification of Diseases-10 (ICD-10) code A04.72 (‘Enterocolitis due to *Clostridium difficile*, not specified as recurrent’). Anti-IL-23 therapy was defined as receipt of any available anti-IL-23 treatment (ustekinumab, guselkumab, tildrakizumab, risankizumab, and mirikizumab).

Baseline demographic, comorbidity (based on ICD-10 codes) and laboratory data were collected, and two-tailed t-tests were used to determine the statistical differences between groups. To ensure comparability between treatment groups, we performed 1:1 propensity score matching based on age and sex. These variables were selected because they are well-established confounders in CDI outcomes, including mortality risk, and were reliably recorded across centers within the TriNetX database.^10,11^ The primary outcome was all-cause death (as recorded in the medical record system from contributing sites) within a 30-d follow-up period following the initial *C. difficile* infection diagnosis. The Kaplan–Meier method was used to measure survival curves. In order to account for patients who exited the cohort during the follow-up period, patients were censored from the survival analysis following the last fact in their record. Use of de-identified, aggregate data was determined non-human subjects research (IRB-Non-HSR 22282) by the UVA Institutional Review Board for Health Sciences Research.

### Data availability

Data displayed and analyzed by the TriNetX Platform are in aggregate form, or any patient-level data are de-identified due to protected health information. A detailed report of the TriNetX query and analysis (including propensity score density function plots before and after matching) are provided in the Supplementary Material. Contact R.P. (saskia.preissner@charite.de) for original data upon reasonable request.

## Results

**Figure 1.**
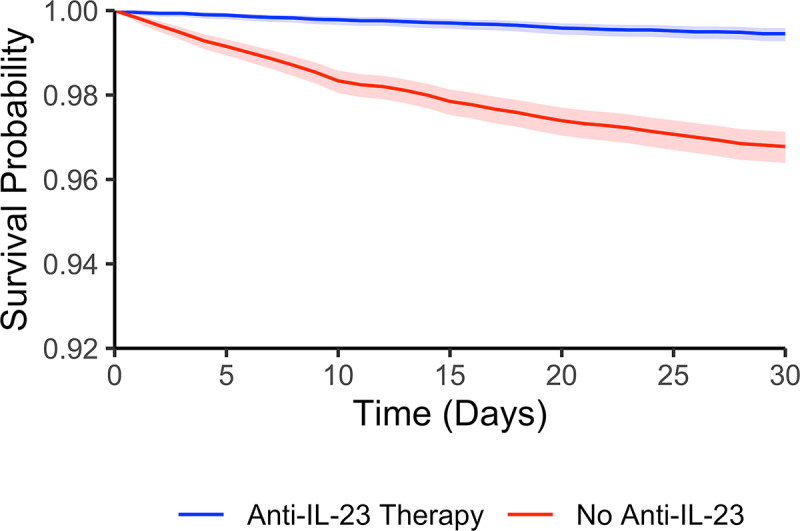
Kaplan–Meier survival curve.

A total of 94 healthcare organizations (100%) responded to the query, with a total of 1,006,866 hospitalized *C. difficile* cases, 9,302 of which had anti-IL-23 medication (ustekinumab, guselkumab, or risankizumab) and 997,564 without anti-IL-23. Baseline characteristics, laboratory measurements, and comorbid conditions for the full cohort (before propensity matching) are shown in Table 1. A total of 287/9,301 (3.1%) propensity-matched control *C. difficile* patients died within the 30-d follow-up period compared to 50/9,301 (0.54%) patients on at least one anti-IL-23 medication. The odds ratio for 30-d mortality in the anti-IL-23 group was 0.17 (95% confidence interval 0.126–0.230). A Kaplan–Meier curve is shown in Figure 1, demonstrating significantly higher survival among anti-IL-23-treated patients (Log-rank *p* < 0.001).

Shaded areas represent the 95% confidence interval.

**Table 1.** Baseline characteristics of the full cohort.

	Controls	Anti-IL-23	*P* value
**(A) Full Cohort**	n = 996,414	n = 9,301	
**Demographics**			
Age (mean±SD)	58.7 ± 20.8	43.2 ± 18.5	<0.001
Female Sex	526,595/996,414 (53.2)	5,238/9,301 (56.3)	<0.001
Race: Non-White	108,946/925,909 (11.8)	853/8,865 (9.6)	<0.001
Ethnicity: Hispanic or Latino	237,021/925,909 (25.6)	1,581/8,865 (17.8)	<0.001
**Comorbidities**			
Inflammatory Bowel Disease (ICD-10 K50-K52)	271,079/957,667 (28.3)	8,412/9,076 (92.7)	<0.001
Psoriasis (ICD-10 L40)	18,739/957,667 (1.96)	1,987/9,076 (21.9)	<0.001
Diabetes (ICD-10 E08-E13)	279,473/925,909 (30.2)	1,711/8,865 (19.3)	<0.001
Chronic Respiratory Disease (ICD-10 J40-J4A)	271,657/925,909 (29.3)	2,714/8,865 (30.6)	0.008
Cancer (ICD-10 C00-D49)	394,073/925,909 (42.6)	4,366/8,865 (49.2)	<0.001
**Laboratory Measurements**	(Non-missing n)	(Non-missing n)	
White Blood Cell Count (x10^9^ cells/mL, mean±SD)	13.4 ± 42.3 (353,899)	10.8 ± 110 (8,416)	<0.001
Creatinine (mg/dL, mean±SD)	1.34 ± 3.1 (743,106)	1.16 ± 5.8 (8,169)	<0.001
Lactate (mmol/L, mean±SD)	1.51 ± 1.26 (340,938)	1.29 ± 0.674 (3,533)	<0.001
Albumin (mg/dL, mean±SD)	3.45 ± 0.78 (715,511)	3.8 ± 0.65 (8,166)	<0.001
**(B) Propensity-Matched Cohort**	n = 9,301	n = 9,301	
Age (mean±SD)	43.2 ± 18.5	43.2 ± 18.5	1
Female Sex	5,238 (56.3)	5,238 (56.3)	1

Data shown as n/non-missing (%) unless otherwise specified.

Abbreviations: SD, standard deviation; International Classification of Diseases-10 code (ICD-10).

Given the possibility of confounding related to higher prevalences of psoriasis and inflammatory bowel disease in the non-anti-IL-23 group, a sensitivity analysis was performed whereupon propensity matching was performed upon the presence of psoriasis and inflammatory bowel disease ICD-10 codes (L40 and E08-E13, respectively) in addition to age/sex and we found similar results (n in propensity matched groups = 9,076; mortality odds ratio = 0.248 (95% confidence interval 0.177–0.347).

## Discussion

To our knowledge, this is the first observational study for the clinical use of host-directed immunotherapy in *C. difficile* infection, suggesting that concomitant anti-IL-23 monoclonal antibodies may significantly reduce *C. difficile* infection mortality. Importantly, by enrolling patients on anti-IL-23 treatment strongly enriched those patients for its two major indications, inflammatory bowel disease (92.7%) and psoriasis (21.9%). *C. difficile* infection is not only more common with inflammatory bowel disease but also more deadly,^12^ and psoriasis patients may be more likely to receive immunosuppressive medications that put them at higher risk for severe *C. difficile* infection. However, traditional baseline *C. difficile* severity markers^3^ (white blood cell count and creatinine) were both significantly lower in the anti-IL-23 group, suggesting that *C. difficile* on anti-IL-23 treatment may have been milder from the onset.

IL-23 inhibition offers several possible advantages and a better safety profile compared to conventional anti-*C. difficile* antibiotics, stool transplant, or surgery. Anti-IL-23 monoclonal antibodies have been in wide clinical use for over a decade and generally considered safe, with mostly benign side effects including nasopharyngitis, upper respiratory tract infection, and injection site erythema.^13^ Antibiotic exposure is the most important predisposing factor for *C. difficile* infection and anti-IL-23 monoclonal antibodies could spare patients from developing further antimicrobial resistance and microbiome disruption. Immunotherapy also does not risk introducing deadly nosocomial pathogens as from a stool transplant.^14^

This study has important limitations. As an observational study, there are likely sources of bias associated with anti-IL-23 treatment that were not captured or adjusted for in our analysis such as time-varying factors or a higher incidence of diabetes among non-anti-IL-23 patients. By virtue of selecting patients on anti-IL-23 therapy, the results may not be generalizable to other patient populations. In addition, identifying deaths directly attributable to *C. difficile* is notoriously difficult,^15^ and, therefore, we chose a relatively short (30-d) follow-up period in order to minimize deaths due to causes other than *C. difficile* infection. Another key limitation was that our CDI definition included both ICD coding and laboratory confirmation. While billing codes are a reasonable proxy for identifying clinically relevant hospitalized *C. difficile* infection using large administrative databases such as TriNetX,^16^ they are subjective. In addition, relying solely on PCR/EIA testing presents its own challenge, as positive results do not necessarily distinguish between *C. difficile* colonization and active infection.

While the TriNetX database is one of the largest cohorts available to study outcomes of *C. difficile* infection, a major drawback is the lack of access to granular patient-level information and missing or heterogeneous data across organizations. As a result, confounding may have occurred due to unmeasured baseline characteristics among the matched control subcohort. Recurrent infection is a major issue with *C. difficile* infection, but this outcome could not be meaningfully examined for two main reasons: the constraints of the TriNetX database in identifying time-varying endpoints besides mortality, and the poor accuracy of retrospective methods besides clinician chart review for identifying recurrence episodes.^17^

Anti-IL-23 is an attractive non-antibiotic, non-surgical candidate treatment for *C. difficile* infection based on our knowledge of *C. difficile* immunopathogenesis and over a decade of clinical experience with anti-IL-23 monoclonal antibodies. This retrospective study further supports the notion that IL-23 inhibition early in the course of *C. difficile* infection to prevent mortality is plausible and further human clinical trials are warranted.

## Supplementary Material

Supplementary_Material.docx
